# An evolved xylose transporter from *Zymomonas mobilis *enhances sugar transport in *Escherichia coli*

**DOI:** 10.1186/1475-2859-8-66

**Published:** 2009-12-15

**Authors:** Chuan Ren, Tingjian Chen, Jingqing Zhang, Ling Liang, Zhanglin Lin

**Affiliations:** 1 Department of Chemical Engineering, Tsinghua University, One Tsinghua Garden Road, Beijing 100084, PR China; 2 Department of Chemical Engineering, Massachusetts Institute of Technology, Cambridge, MA 02139, USA

## Abstract

**Background:**

Xylose is a second most abundant sugar component of lignocellulose besides glucose. Efficient fermentation of xylose is important for the economics of biomass-based biorefineries. However, sugar mixtures are sequentially consumed in xylose co-fermentation with glucose due to carbon catabolite repression (CCR) in microorganisms. As xylose transmembrance transport is one of the steps repressed by CCR, it is therefore of interest to develop a transporter that is less sensitive to the glucose inhibition or CCR.

**Results:**

The glucose facilitator protein Glf transporter from *Zymomonas mobilis*, also an efficient transporter for xylose, was chosen as the target transporter for engineering to eliminate glucose inhibition on xylose uptake. The evolution of Glf transporter was carried out with a mixture of glucose and xylose in *E. coli*. Error-prone PCR and random deletion were employed respectively in two rounds of evolution. Aided by a high-throughput screening assay using xylose analog *p*-nitrophenyl-*β*-D-xylopyranoside (pNPX) in 96-well plates, a best mutant 2-RD5 was obtained that contains several mutations, and a deletion of 134 residues (about 28% of total residues), or three fewer transmembrane sections (TMSs). It showed a 10.8-fold improvement in terms of pNPX transport activity in the presence of glucose. The fermentation performance results showed that this mutant improved xylose consumption by 42% with M9 minimal medium containing 20 g L^-1 ^xylose only, while with the mixture sugar of xylose and glucose, 28% more glucose was consumed, but no obvious co-utilization of xylose was observed. Further glucose fed-batch experiments suggested that the intracellular metabolism of xylose was repressed by glucose.

**Conclusions:**

Through random mutagenesis and partial deletion coupled with high-throughput screening, a mutant of the Glf transporter (2-RD5) was obtained that relieved the inhibition of xylose transport by glucose. The fermentation tests revealed that 2-RD5 was advantageous in xylose and glucose uptakes, while no obvious advantage was seen for xylose co-consumption when co-fermented with glucose. Further efforts could focus on reducing CCR-mediated repression of intracellular metabolism of xylose. Glf should also serve as a useful model to further exploit the molecular mechanism of xylose transport and the CCR-mediated inhibition.

## Background

Currently, most of the industrial materials and fuels are made from petroleum, which is irreversibly being depleted. Lignocellulosic biomass, the most abundant raw material from hardwood, softwood, agricultural residues and grasses, is seen as an enormous potential feedstock for future production of valuable chemicals and biofuels [1-3]. In biorefinery, several bacteria, yeasts, and fungi have been used for processing lignocellulosic biomass into various industrial chemicals such as ethanol, succinic acid, lactic acid, butanol and acetone [4-6]. D-Xylose is a second most abundant sugar component of lignocellulose besides glucose, and comprises up to 25% of the total sugar content in lignocellulosic hydrolysates [7-9]. However, sugar mixtures are sequentially consumed in fermentation due to carbon catabolite repression (CCR) in microorganisms [10], which significantly reduce productivity and efficiency of the biorefinery processes. Therefore, the efficient co-utilization of xylose with glucose is a prerequisite for large-scale production of biofuels and biochemicals.

In *Escherichia coli*, regulation of CCR is brought about by the modulation of the phosphorylation state of EIIA^Glc^, which is encoded by the *ccr *gene and an IIA component of the glucose-specific phosphoenolpyruvate-carbohydrate phosphotransferase system (PTS). In the presence of glucose, EIIA^Glc ^is preferentially dephosphorylated and binds to non-PTS permeases and also down-regulate genes related to metabolism of non-PTS sugars, including xylose [10,11]. Previously, components of the PTS or related systems have been knocked out to reduce glucose inhibition [12-16], but with mixed outcomes [13]. We sought a different approach by firstly modifying xylose transporters to reduce glucose inhibition on xylose uptake.

Glucose facilitator protein Glf transporter (encoded by the *glf *gene) from *Zymomonas mobilis *belongs to the major facilitator superfamily (MFS) class of proteins [17]. It is a low-affinity, high-velocity carrier which also transports fructose, xylose in addition to glucose. This type of facilitators functions without additional energy but only relies on the cross-membrane concentration gradients of sugars, and thus is energetically more efficient. Glf transporter also can take up xylose rapidly with a maximum rate twice that for glucose at 5°C [18], although glucose is still utilized preferentially when co-fermenting with xylose for a recombinant xylose-fermenting strain of *Z. mobilis *CP4(pZB5) [19]. Furthermore, this protein has been functionally expressed in *E. coli *strains for transporting glucose and fructose [20]. And in our previous work, it has been shown that the xylose transport activity of Glf transporter was totally inhibited in the presence of glucose in *E. coli *[21]. Taken together, Glf transporter is a useful transporter for studying the glucose inhibition and for creating improved xylose carrier variants with enhanced activity in the presence of glucose.

Although there is no report about the structure of the Glf transporter, lactose permease (LacY) from *E. coli*, which belongs to the same MFS class of proteins, has been studied in detail. LacY is a cytoplasmic membrane protein with 12 hydrophobic transmembrane α-helical domains with the N- and C- termini on the cytoplasmic face of the membrane. A study on LacY revealed that several residues in its cytoplasmic loops IV/V and VI/VII played an important role in the binding of unphosphorylated EIIA^Glc ^[16]. This provides important clues for engineering of Glf transporter.

In this study, we proposed to eliminate the interaction between Glf transporter and EIIA^Glc ^in *E. coli *by manipulating Glf transporter directly. Error-prone PCR [22,23] and random deletion [24-26] strategies were employed in two rounds of directed evolution respectively to create Glf transporter libraries. Existing analyses of xylose uptake were commonly involved in utilizing isotopic substrates or high-performance liquid chromatography (HPLC) [20,27,28], which were not the appropriate methods for screening due to complicated operations. We recently developed a high-throughput screening method for xylose transporter assay *in vivo *by co-expressing xylosidase from *Bacillus pumilus *(XynB) with Glf transporter, which was able to utilize a commercially available xylose analog, *p*-nitrophenyl-*β*-D-xylopyranoside (pNPX) [21]. From the Glf transporter mutant libraries screened with this XynB-mediated assay in 96-well plates, a successful mutant was obtained which was 10.8-fold more efficient than the wild type in transporting xylose in LB medium supplemented with 20 g L^-1 ^glucose while the xylose transport activity of the wild type was totally inhibited by glucose. The fermentation performance of this mutant in the presence of xylose and/or glucose was also evaluated and discussed.

## Results and Discussion

### First round evolution of Glf transporter

In the first round of directed evolution, error-prone PCR was used to engineer Glf transporter. The template pET30a-*glf-xynB *was constructed as previously reported, and 0.15 mM Mn^2+ ^was added following the standard error-prone PCR protocol. The library was transformed into *E. coli *BL21(DE3). 3000 variants were screened using the XynB-mediated high-throughput screening method in 96-well plate [21], and a best mutant 1-6C7 was obtained. It showed about one-fold improvement in transporting pNPX compared with the wild type in LB medium supplemented with 20 g L^-1 ^glucose, while there was no statistically significant difference between its transport activity and that of wild type in the absence of glucose (p = 0.509) (Table 1). Sequencing showed that it contained six amino acid mutations compared with wild type Glf (H215Q, T242W, D251V, Q279R, N427H and G469W).

**Table 1:** whole cell pNPX transport assay results for wild type Glf and its mutants.

BL21(DE3)/pET30a-glf-xynB	Whole cells assay (nmol min^-1 ^mg dw^-1^)		p value	
	
	LB	LB/Glc	LB	LB/Glc
wild type	14.6 ± 2.0	0.6 ± 0.2	-	-
1-6C7	12.9 ± 0.3	1.2 ± 0.1	0.509	0.035
2-RD5	17.8 ± 2.6	7.1 ± 0.8	0.406	0.009

LB/Glc, LB medium supplemented with 20 g L^-1 ^glucose; the standard deviations shown above were from triplicate independent cultures.

### Second round evolution of Glf and secondary structure prediction by HMMTOP

**Figure 1:**
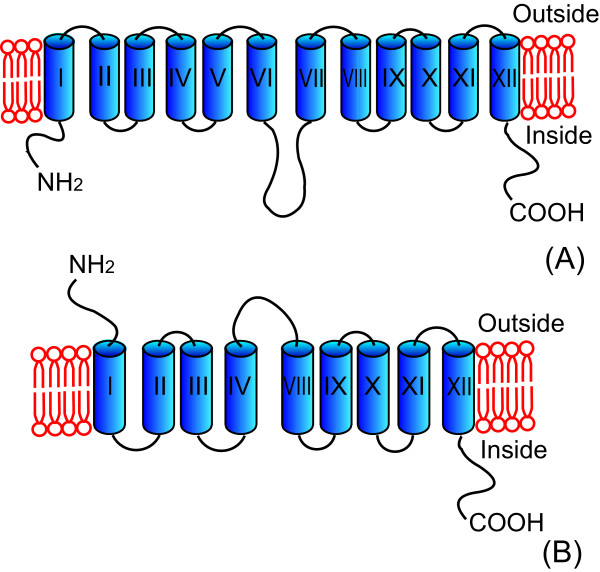
**Secondary structure models of the wild type Glf and the mutant 2-RD5 predicated by HMMTOP**. (A) Wild type Glf has 12 TMSs and both N- and C- termini are inside the cytoplasm. (B) Mutant 2-RD5 has 9 TMSs and N-terminus is on the outside of the cytoplasm, while C-terminus is on the inside of the cytoplasm.

**Figure 2:**
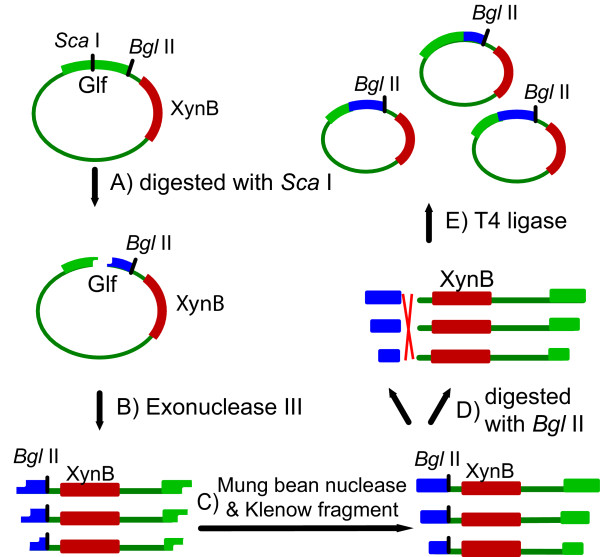
**Schematic overview of random deletion used in this study**. A) Linearization of the plasmid by restriction digestion at *Sca *I site. B) Random deletion with Exo III. C) Blunt-ended with mung bean nuclease and Klenow fragment. D) Single digested with *Bgl *II. E) Random ligation with T4 DNA ligase.

While the improvement in the first round of evolution was significant (p = 0.035), it was insufficient for our purpose. We thus took a more focused approach by carefully examining the Glf loops. The study on *E. coli *lactose permease (LacY), a close relative of Glf, suggested several possible residues in its cytoplasmic loops targeted by the unphosphorylated EIIA^Glc ^[16], an essential step for the CCR effect. This prompted us to carefully examine the Glf loops. As the structure of Glf transporter was unknown, we predicted its secondary structures by HMMTOP [29-31]. As shown in Figure 1A, the model suggested that Glf had 12 transmembrane sections (TMSs) and both N- and C- termini were in the cytoplasm. Corresponding amino acid residues for the 12 TMSs were 13-35, 52-73, 86-105, 124-146, 159-176, 197-218, 258-277, 304-322, 335-354, 367-386, 399-417 and 434-451. The large loop between helices VI and VII were subsequently chosen for random deletion. To this end, *Sca *I site was introduced in the loop using pET30a-*glf(1-6C7)*-*xynB *as template. The protocol was designed as such that the two Glf DNA fragments progressively deleted from the point of *Sca *I were then mixed randomly again in re-ligation, by extracting the second set of DNA fragments from the linearized plasmid (Figure 2D) and then ligated back into the same plasmid (Figure 2E). This created a library of Glf variants that were truncated randomly at either end of the reference point of the *Sca *I site. A total of 2350 variants were screened by the XynB method with 20 g L^-1 ^glucose in the medium in 96 well plates, and good mutants were confirmed by growing the cells in test tubes. A best mutant, 2-RD5, was obtained, which showed a 10.8-fold improvement (p = 0.009) in terms of pNPX transport activity in the presence of glucose. In the LB medium alone, the pNPX transport activity of 2-RD5 was also slightly higher than that of the wild type (Table 1), although this increase was not statistically significant (p = 0.406). Sequencing revealed it had about a deletion of 134 residues (about 28% of the total residues) and contained one additional amino acid mutation (R336S) compared with the template 1-6C7. According to the model predicted by HMMTOP (Figure 1B), TMSs V, VI and VII were completely deleted together with parts of the loops between IV/V and VII/VIII in mutant 2-RD5, and the N-terminus was now on the outside of the cytoplasm, while the C-terminus was on inside of the cytoplasm. It should also be noted that only two of the original mutations in the template 1-6C7 remained in 2-RD5. The functions of these three point mutations (including the additional mutation generated during random deletion) require further dissection.

### Characterization of xylose transport activity of 2-RD5 in the presence of glucose

**Figure 3:**
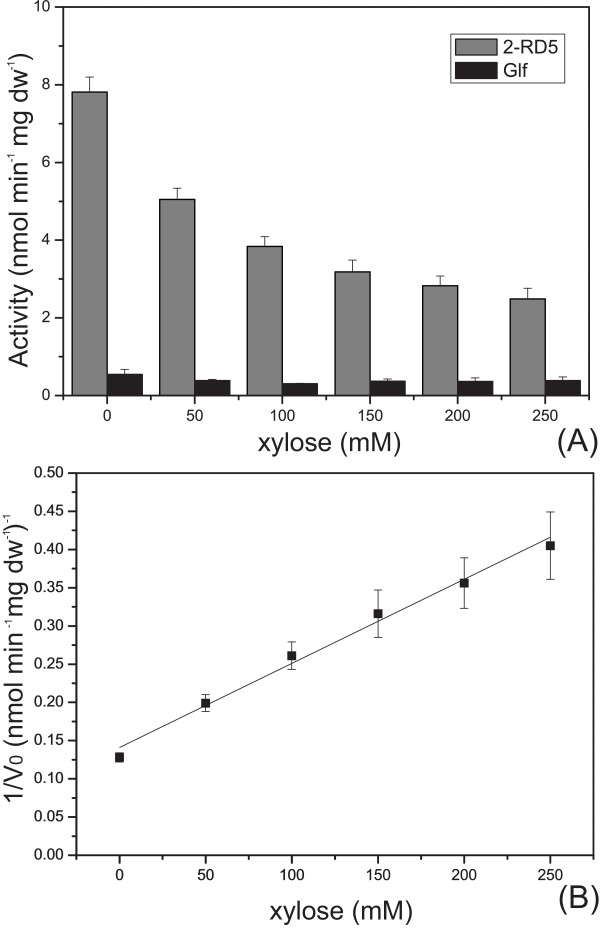
**Xylose inhibition assay for Glf mutant 2-RD5**. (A) Xylose inhibition of pNPX transport (normalized by dry cell weight) for BL21(DE3)/pET30a-*glf(2-RD5)-xynB *(gray columns) and BL21(DE3)/pET30a-*glf-xynB *(black columns). The inhibition assay was done in the presence of 2% glucose. (B) Relationships between the invert of the initial velocity of pNPX transport and the concentrations of xylose for BL21(DE3)/pET30a-*glf(2-RD5)-xynB*. All data represent triplicate measurements of same cultures.

In the previous work [21], we showed that xylose and its analog pNPX were competing substrates in terms of Glf transport, and thus the two transport activities were correlated. To quantitatively correlate the transport behaviours of xylose and its analog pNPX for the best mutant 2-RD5, similarly inhibition experiments were again carried out, but in the presence of glucose. The whole cell pNPX transport activity was measured in the presence of different concentrations of xylose. As shown in Figure 3B, for mutant 2-RD5 in the presence of glucose, the inhibition by xylose still matched the classical Michaelis-Menten equation (the R^2 ^value was 0.990). In contrast, for the wild type in the presence of glucose, the transport activity of pNPX was approximately constant in different concentration of xylose, and close to the background (about 2 nmol min^-1 ^mg dw^-1^) (Figure 3A), indicating an almost total inhibition by glucose. These results showed that the mutant 2-RD5 significantly improved xylose transport activity in the presence of glucose.

### Fermentation performance of 2-RD5

**Figure 4:**
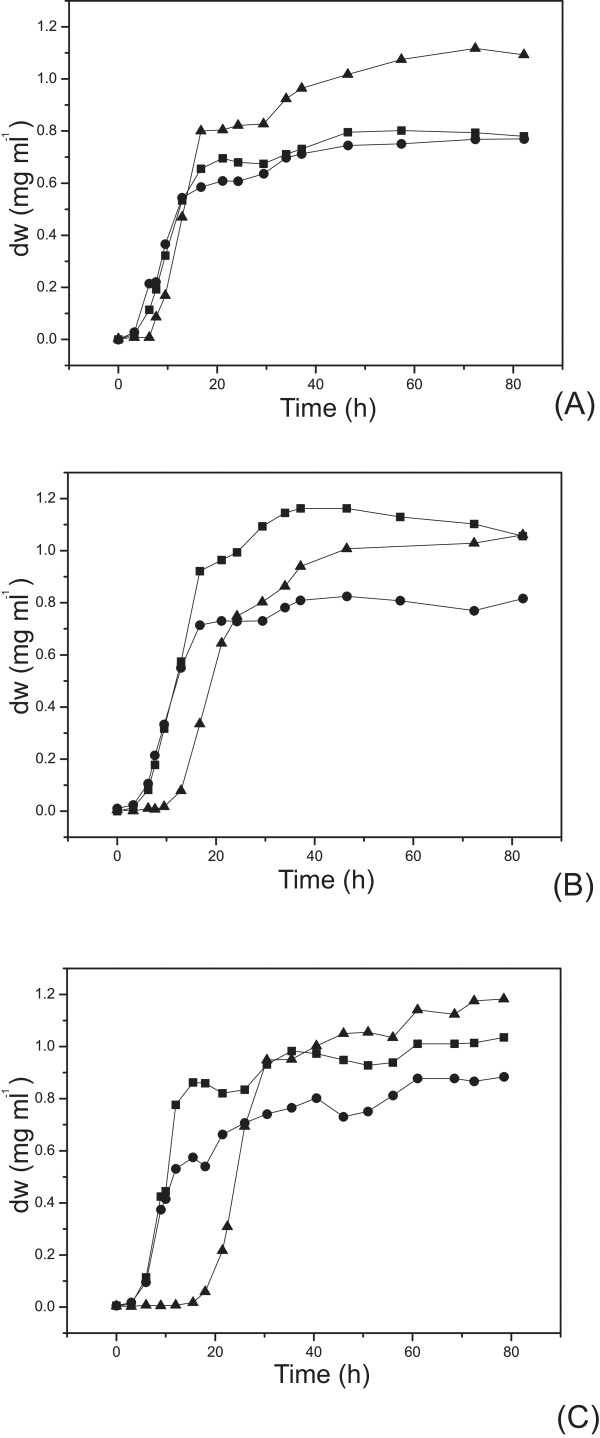
**Growth curves of *E. coli *BL21(DE3) cells containing BL21(DE3)/pET30a-*glf(2-RD5)*-*xynB*, BL21(DE3)/pET30a-*glf*-*xynB *and BL21(DE3)/pET30a**. (A) in M9 minimal medium supplemented with 20 g L^-1 ^xylose. (B) in M9 minimal medium supplemented with 10 g L^-1^glucose and 10 g L^-1 ^xylose. (C) in M9 minimal medium supplemented with 20 g L^-1 ^xylose, adding 5 g L^-1 ^glucose after 1 h IPTG induction. *E. coli *BL21(DE3)/pET30a (filled black square), wild type *E. coli *BL21(DE3)/pET30a-*glf*-*xynB *(filled black circle) and *E. coli *BL21(DE3)/pET30a-*glf(2-RD5)*-*xynB *(filled black triangle).

**Figure 5:**
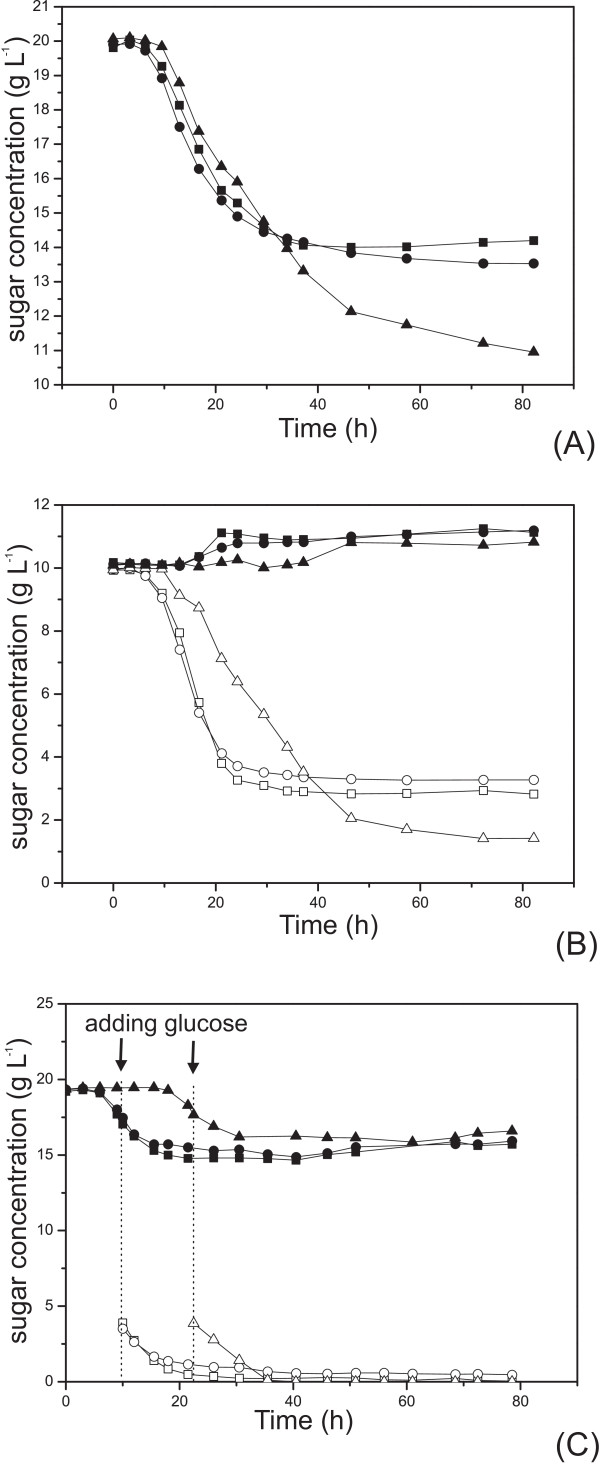
**Sugar consumption in flask fermentation**. (A) in minimal medium supplemented with 20 g L^-1 ^xylose, B) in minimal medium supplemented with 10 g L^-1 ^glucose and 10 g L^-1 ^xylose, C) in M9 minimal medium supplemented with 20 g L^-1 ^xylose, adding 5 g L^-1 ^glucose after 1 h IPTG induction. Sugars for different strains are: xylose of *E. coli *BL21(DE3)/pET30a (filled black square), xylose of wild type *E. coli *BL21(DE3)/pET30a-*glf*-*xynB *(filled black circle), xylose of *E. coli *BL21(DE3)/pET30a-*glf(2-RD5)*-*xynB *(filled black triangle), glucose of *E. coli *BL21(DE3)/pET30a (empty square), glucose of wild type *E. coli *BL21(DE3)/pET30a-*glf*-*xynB *(empty circle) and glucose of *E. coli *BL21(DE3)/pET30a-*glf(2-RD5)*-*xynB *(empty triangle).

Consumption of xylose in the presence or absence of glucose for *E. coli *BL21(DE3) cells harbouring pET30a-*glf(2-RD5)*-*xynB *were evaluated at 37°C, and normalized by cell dry weight. BL21(DE3)/pET30a and BL21(DE3)/pET30a-*glf*-*xynB *were monitored simultaneously as controls. Typical fermentation results are shown in Figures 4 and 5. After cultivated in M9 minimal medium supplemented with 20 g L^-1 ^xylose for over 82 hours, 9.1 g L^-1 ^xylose was consumed, about 42% more than the control BL21(DE3)/pET30a-*glf*-*xynB *(Figure 5A), which was significantly higher than the standard deviation of 1.5% observed for xylose consumption in this study. Consistently, BL21(DE3)/pET30a-*glf(2-RD5)*-*xynB *produced 41% more biomass (Figure 4A). On the other hand, when minimal medium supplemented with 10 g L^-1 ^glucose and 10 g L^-1 ^xylose was used, BL21(DE3)/pET30a-*glf(2-RD5)*-*xynB *consumed 8.5 g L^-1 ^glucose at 82 hours, about 27% more than the control BL21(DE3)/pET30a-*glf*-*xynB *(Figure 5B), and 30% more biomass was produced (Figure 4B). These results showed that the Glf transporter mutant 2-RD5 enhanced its sugar transport property, both for xylose (in the presence of xylose alone) and glucose. However, in the minimal medium supplemented with 10 g L^-1 ^xylose and 10 g L^-1 ^glucose, there was no obvious consumption of xylose for all of the strains, including BL21(DE3)/pET30a-*glf(2-RD5)*-*xynB*. This was in direct contradiction to the greatly improved xylose transport in the presence of glucose, demonstrated by the pNPX assay. A plausible explanation might be that while the inhibition of xylose transport by glucose was relieved, the inhibition of intracellular xylose metabolism mediated by glucose now became the rate-limiting step.

A set of glucose-fed batch cultivation experiments was done to verify the metabolism inhibition of xylose. The three strains were first inoculated in the minimal medium with 20 g L^-1 ^xylose alone, IPTG was added to induce the expression of the transporter when the OD reached 0.6, and then 5 g L^-1 ^glucose was added after 1 hour induction. As shown in Figure 5C, when glucose was supplemented, xylose utilization was gradually inhibited in all three strains. After 8~10 hours, the xylose consumption was essentially ceased. These results suggested that glucose supplementation affected the downstream xylose metabolism and gradually blocked the xylose utilization, Consistent with the co-fermentation results (Figure 5B), BL21(DE3)/pET30a-*glf(2-RD5)*-*xynB *almost exhausted glucose in the medium, about 28% more than BL21(DE3)/pET30a-*glf*-*xynB *(Figure 5C), and produced 34% more biomass (Figure 4C).

The fermentation results showed that, regardless what carbon sources were used, BL21(DE3)/pET30a-*glf(2-RD5)*-*xynB *consumed more sugars and produced more biomass. However, in all cases it showed a significant lag phase (Figure 4A-C), for which the reason is unclear. It might be that the cells need longer time to adapt to this mutant transporter during growth. It was also interesting to note that *E. coli *BL21(DE3)/pET30a-*glf(2-RD5)*-*xynB *was lighter than other two strains in the equal OD_600 _(The unit cell dry weight of BL21(DE3)/pET30a, BL21(DE3)/pET30a-*glf*-*xynB *and BL21(DE3)/pET30a-*glf(2-RD5)*-*xynB *were 0.345, 0.340 and 0.247 mg OD^-1 ^respectively), and the size of the cells was also slightly smaller than others judging from microscopic observations.

## Conclusions

Several research groups have deleted components of PTS or related systems to reduced glucose inhibition [12-16]. As these components such as EIIA^Glc ^take part in other cellular pathways, simple knock out may lead to unexpected effects [16]. We thus feel that engineering a transporter less sensitive to glucose represents a useful first step toward resolving CCR. In this work, through random mutagenesis and partial deletion coupled with a high throughput screening assay, a mutant of the Glf transporter (2-RD5) was obtained that showed a much improved xylose transport activity than the wild type in the presence of glucose. The fermentation tests revealed that 2-RD5 was advantageous in xylose and glucose uptake. While no obvious advantage in xylose consumption was observed when co-fermented with glucose, this could be attributed to the inhibition on the intracellular xylose metabolism exerted by glucose, and thus further engineering to relieve this intracellular aspect of the CCR mechanism would eventually lead to efficient co-utilization of xylose with glucose in microorganisms.

Mutant 2-RD5 is much shorter than we had anticipated, a total of 134 residues were deleted, representing TMSs V, VI and VII, and parts of the loops between IV/V and VII/VIII. This presumably affects the binding of unphosphorylated EIIA^Glc ^to Glf, as loops IV/V and VI/VII have been found to be involved in the association of EIIA^Glc ^to a related transporter LacY [16]. Moreover, according to the prediction by HMMTOP, the orientation of first four TMSs in 2-RD5 is also inverted such that now the N-terminus is flipped to the outside of the cytoplasm. A literature search suggests that the majority of transporters consist of TMSs between 10 and 14 while some others have fewer TMSs, and those with fewer than 10 TMSs are probably present in the membrane as dimers [32]. As an example, the cardiac Na^+^-Ca^2+ ^exchanger (NCX1), a membrane protein that extrudes Ca^2+ ^from cells using the energy of the Na^+ ^gradient has a similar structure as the predicted one for 2-RD5. It consists of nine TMSs with a large intracellular loop between TMSs V/VI and two re-entrant loops connecting TMSs II/III and TMSs VII/VIII, and its N-terminus is on the outside of the cytoplasm, while the C-terminus is on the inside of the cytoplasm [33,34]. NCX1 has been suggested to form a dimer in the membrane [35]. Whereas the actual structure and mechanism of 2-RD5 requires further study, we presume the functional structure of 2-RD5 might be similar to NCX1.

In future work, Glf should serve as a useful model to further exploit the molecular mechanism of xylose transport and CCR-mediated inhibition. Random or more focused mutagenesis, as well as structural analysis, of this transporter should continue to reveal useful clues, which will lead to construction of an efficient transporter for xylose.

## Methods

### Materials

Restriction enzymes, DNA-modifying enzymes, and DeepVent^® ^polymerase were from New England Biolabs (Ipswich, MA, USA), *Taq *DNA polymerase from Takara (Dalian, China), and *Pfu *DNA polymerase from Tiangen (Beijing, China). The kits for DNA purification, gel recovery and plasmid mini-prep were either from Tiangen or QIAgen (Valencia, CA, USA). Isopropyl *β*-D-thiogalactoside (IPTG), and DNase I were from TaKaRa (Dalian, China). pNPX was obtained from Sigma-Aldrich (St. Louis, MO, USA). Oligonucleotides were synthesized by TaKaRa (Dalian, China) or by Sunbiotech Co., Ltd. (Beijing, China). Sequence analysis was performed either by Sangon (Shanghai, China) or by Sunbiotech. All other chemicals were of the highest grade available and were obtained from standard commercial sources. *E. coli *BL21(DE3) and plasmid pET30a(+) were obtained from Novagen (Wisconsin, USA). The plasmid pET30a-*glf-xynB *was constructed previously in this laboratory [21].

### Construction of random mutant library by error-prone PCR

The *glf *gene was randomly mutated by a standard error-prone PCR protocol with 0.15 mM Mn^2+ ^[36], The primers were as follows: forward primer epGLF, 5'-CGTATTA**TGGCCA**TTGTGACGGGTGC-3'; reverse primer GLF^DOWN^, 5'-AAGACCG**AGATCT**CTACTTCTGGGAGCGCCACAT-3' (*Msc *I and *Bgl *II sites are bold, respectively), and then subcloned into the *Msc *I - *Bgl *II sites in pET30a-*glf-xynB*. The resulting random mutant library was transformed into *E. coli *BL21(DE3) for the followingscreening.

### Site-directed mutagenesis

Before the second round of evolution, *Sca *I site was firstly inserted into the best mutant (1-6C7) screened from the first round of directed evolution on Glf transporter in plasmid pET30a-*glf(1-6C7)-xynB *by site-directed mutagenesis. Overlapping PCR were carried out using the following primers, epGLF and ScaI^1^, 5'-TCAGATTAGGA**AGTA**CTTGCGGTTCCAGACG-3' for reaction I; and GLF^DOWN ^and ScaI^2^, 5'-GGAACCGCAAG**TACT**TCCTAATCTGATGA-3' for reaction II (mutated positions are bold). The amplified DNA fragments of reaction I and II were treated with *Dpn *I respectively, and then overlapped for 10 cycles under the following conditions: 94°C for 1 min, 70°C for 1 min, and 72°C for 2 min. The full length mutated gene was amplified with primers epGLF and GLF^DOWN ^under 20 cycles of 94°C for 1 min, 63°C for 1 min, and 72°C for 2 min, and inserted into the *Msc *I - *Bgl *II sites in pET30a-*glf*-*xynB *to yield pET30a-*glf(1-6C7)*-*xynB*-ScaI.

### Construction of random mutant library by random deletion

Nine micrograms of *Sca *I-digested pET30a-*glf(1-6C7)*-*xynB*-ScaI were mixed with 15 μL of 10× NEBuffer 1 (New England Biolabs) and the volume was adjusted to 150 μL with deionized distilled water. The solution was equilibrated at 12°C. At time zero, 900 U Exonuclease III (Exo III) were added. 5 μL samples were removed every 30 sec thereafter for 15 min and added to a tube, and heated at 70°C for 20 min to fully inactivate Exo III (Figure 2B). After all the samples were collected, the single-stranded 5'-overhangs were removed upon incubation with 9 U mung bean nuclease at 30°C for 30 min. The reaction was quenched with 0.01% SDS, and the DNA was purified. To facilitate the re-ligation (see below), the purified DNA was blunt-ended with 3.2 U Klenow fragment in 10× NEBuffer 2 (New England Biolabs) and dNTPs (final concentration 33 μM each nucleotide) for 15 min at 25°C. Then EDTA was added to a final concentration of 10 mM, and the tube was incubated at 75°C for 20 min (Figure 2C). The purified DNA was then singly digested with *Bgl *II and extracted to obtain a progressively truncated library of fragments (Figure 2D). These fragments were then ligated back into the plasmid.

### 96-well plate assay for pNPX transport activity

*E. coli *BL21(DE3) cells harbouring the Glf library of the first or second round evolution were inoculated in LB medium (200 μL) supplemented with kanamycin (50 μg mL^-1^) in 96-well microtiter plates. The plates were grown at 37°C overnight; 5 μL of each culture was used to inoculate plates with 200 μL LB/kanamycin medium or 200 μL LB/kanamycin medium with glucose (20 g L^-1^) in each well for expression. After 1.5 h of growth at 37°C, IPTG was added to a final concentration of 1 mM, and protein expression was carried out at 37°C for 4 h. For the whole cell assay, 85 μl of cell culture was added into a mixture of 15 μl of 6.25 mM pNPX and 100 μl of LB medium. XynB was able to cleave the xylose analog pNPX to xylose and a chromogenic group *p*-nitrophenol (pNP). Reactions were incubated at 37°C in the chamber of a SpectroMAX 190 Microtiter reader (Molecular Devices, CA), and absorbance at 405 nm was measured at 30 sec intervals for 30 min. The pNPX transport activity was normalized against the respective dry cell weight. The concentration of pNP generated was calculated using a molar extinction coefficient of 18,700 M^-1 ^cm^-1 ^[37]. No detectable XynB activity was found in the supernatants of the cultures of *E. coli *BL21(DE3) cells harbouring pET30a-*glf*-*xynB*, or pET30a-*glf(1-6C7)*-*xynB*, or pET30a-*glf(2-RD5)*-*xynB*.

### Xylose competing inhibition experiment

pNPX uptake activity of whole cell was measured in the presence of different concentration of xylose. *E. coli *BL21(DE3) cells harbouring plasmid pET30a-*glf*-*xynB *or pET30a-*glf(2-RD5)-xynB *was grown at 37°C in LB medium containing kanamycin (50 μg mL^-1^). The saturated overnight cultures were diluted 50-fold into fresh LB/kanamycin medium supplemented with glucose (20 g L^-1^) and grown at 37°C for about 1.5 h to reach an OD_600 _of 0.5~0.6. Protein expression was initiated with 1 mM IPTG, and continued for 4 h at 37°C. After that, 85 μl cell culture was added into a mixture of 15 μL pNPX solution (6.25 mM) and 100 μL LB solution of xylose (0, 100 mM, 200 mM, 300 mM, 400 mM, 500 mM) to initiate the whole cell reaction. The reaction and activity assay was carried out as in previous part.

### Flask fermentation experiments

Single colonies of *E. coli *BL21(DE3)/pET30a-*glf(2-RD5)-xynB*, BL21(DE3)/pET30a-*glf*-*xynB *and BL21(DE3)/pET30a were inoculated into 5 mL M9 minimal medium [38] supplemented with kanamycin (50 μg mL^-1^) and 20 g L^-1 ^xylose, or 10 g L^-1 ^glucose and 10 g L^-1 ^xylose, and grown at 37°C and 250 rpm overnight. Then the cultures were diluted 100-fold in to 100 mL medium in 500-mL flasks, and grown at 37°C. IPTG was added to a final concentration of 0.02 mM when OD_600 _values reached 0.5~0.6. A second set of fermentation experiments were also carried out by adding 5 g L^-1 ^glucose into M9 minimal medium with 20 g L^-1 ^xylose 1 h post IPTG induction to analyze the glucose effect for the fermentation of xylose. At indicated intervals, OD_600 _values of the cultures were recorded, and 1 mL culture was retrieved for determination of the xylose and glucose concentrations using high-performance liquid chromatography (Shimadzu, Kyoto, Japan), using an Aminex ion-exclusion HPX-87H cation-exchange column (BioRad, California, USA) at 55°C with 5 mM H_2_SO_4 _as the mobile phase at a flow rate of 0.5 mL min^-1^. The sugars were detected with a RID-10A refractive index detector (Shimadzu, Kyoto, Japan). All samples for sugar analysis were filtered through 0.22 μm filter membranes before using. For determination of cell dry weight, 40 ml cells were collected at intervals after IPTG induction. The cell pellets were washed with deionized distilled water before drying. Cell dry weight was determined by drying cells in pre-weighed tubes at 65°C for 2 days.

## Lists of abbreviations

For ease of readability, the following abbreviations were used: CCR: carbon catabolite repression; Glf: *Zymomonas mobilis *Glucose facilitator protein; HPLC: high-performance liquid chromatography; IPTG: Isopropyl *β*-D-thiogalactoside; LacY: *Escherichia coli *lactose permease; MFS: major facilitator superfamily; NCX1: The Na^+^-Ca^2+ ^exchanger; pNP: *p*-nitrophenol; pNPX: *p*-nitrophenyl-*β*-D-xylopyranoside; PTS: phosphotransferase system; TMS: transmembrane sections; XynB: *Bacillus pumilus *xylosidase.

## Competing interests

The authors declare that they have no competing interests.

## Authors' contributions

CR carried out the fermentation performance and transport activity characterization, and drafted the manuscript. TC participated in fermentation performance and transport activity characterization. JZ carried out the cloning and the first round of evolution. LL carried out the second round of evolution. ZL conceived the study, designed and supervised the experiments, and revised the manuscript. All authors read and approved the final manuscript.
